# Using *Chironomus dilutus* to identify toxicants and evaluate the ecotoxicity of sediments in the Haihe River Basin

**DOI:** 10.1038/s41598-017-01631-5

**Published:** 2017-05-03

**Authors:** Xiaolei Zhu, Baoqing Shan, Wenzhong Tang, Chao Zhang

**Affiliations:** 1 State Key Laboratory of Environmental Aquatic Chemistry, Research Center for Eco-Environmental Sciences, Chinese Academy of Sciences, Beijing, 100085 P.R. China; 2 University of Chinese Academy of Science, Beijing, 100049 P.R. China

## Abstract

To effectively manage a watershed and successfully restore a river system, it is very important to assess the toxicity of sediments and identify the substances causing the toxicity. Seventy-six sediments collected in the Haihe River Basin (HRB) in China were screened for acute toxicity using *Chironomus dilutus*. We found that sediments from more than 32% of sampling sites, distributed mainly in the Ziya tributary and along the estuary, were acutely toxic to midges. A toxicity identification evaluation showed that the toxicity of the sediment samples was mainly from ammonia nitrogen, metals, and organics. Calculations of the toxic unit (TU) showed that ammonia and metals contributed more to sediment toxicity than organics, and that PAHs may have contributed in other tributaries. A modified three-step sequential extraction procedure to assess the bioavailability of the metals indicated that the toxicity from metals was mainly from Cd and Zn. This is one of the first studies in which this type of approach has been applied to directly connect contaminants with ecological effects in the HRB.

## Introduction

In recent years, contaminated sediments in water bodies worldwide have emerged as a major concern for ecological and human health^1^. Out of a range of factors including habitat change and introduced species, chemical pollution is considered one of the most important influences on the quality of sediment^2^. Effective management of contaminated sediment requires an assessment of the nature and degree of contamination, followed by recommendations for remediation. For the former, various weight of evidence (WOE) approaches have been developed that provide multidisciplinary characterization techniques and assess sediment quality by integrating data from different studies^3–5^. Once the risk zone is screened, the particular approaches for remediating contaminated sediments are chosen^6^. Remediation is usually a very costly process, and should be optimized by including a robust investigation of the origin of the toxicity in complex samples^7, 8^. The toxicity identification evaluation (TIE) is an extremely useful tool that can help identify the main toxicants in sediment^9, 10^.

The TIE approach identifies the main toxicants in environmental samples by integrating bioassays and physical and chemical manipulations in an iterative process that allows the investigator to focus the investigation on the target toxicant^7, 11^. The TIE approach is divided into three phases: characterization into main classes with physical and chemical manipulations (Phase I), identification of the toxicants through analytical techniques and a toxicity evaluation (Phase II), and confirmation of the actual causes of toxicity through collating the corroborating toxicant data (Phase III)^10, 12^. This method has been used successfully for identifying toxicants in both sediment interstitial water and pure sediment^11, 13, 14^. Previous studies have shown that better outcomes are expected from a TIE of aggregated sediment than from observations of intact sediments *in situ* ^10, 15, 16^.

The TIE procedure has been routinely used for environmental risk assessments in North America, Europe, Australia and Brazil^8, 17–19^, where the level of contamination was relatively low and the toxicity in sediment was mainly identified as arising from a single class of contaminants^13, 20^. In China, however, few studies have applied the TIE approach to identify contaminants in sediments^12, 13, 21^. The Haihe River Basin is in one of the most industrialized and urbanized regions in China. The rapid rate of urbanization, which increased from 18% in 1978 to 46% in 2009, has subjected river ecosystems to great stress, especially in the plain areas^22^. The population in the Haihe River Basin increased from 8.16 million in 1985 to about 180 million in 2014, and the industrial wastewater discharge increased from 0.33 billion tons to 2.37 billion tons in the same period. Increases in freshwater consumption and corresponding decreases in rainfall have resulted in water shortages in the Haihe Basin. Furthermore, wastewater discharges from industry have dramatically increased over time, and sewage treatment facilities have become increasingly inadequate, to the point that the Haihe has become the most polluted river in China. Against this backdrop, the objectives of the present study were to (1) assess the biotoxicity of surface sediments from the southern part of the Haihe River Basin using *C. dilutus*, and (2) identify the compounds causing the toxicity in Haihe River sediments using a comprehensive sediment toxicity assessment. This is the first time that this method has been used to describe the nature of the toxicity in the Haihe River, and we hope that this study can provide help in validating the technical applicability of whole-sediment TIE in complex systems, and will be a useful reference for other similar studies of sediment pollution control and management.

## Results and Discussion

### Toxicity screening of sediments in the rivers of Haihe Basin

**Figure 1:**
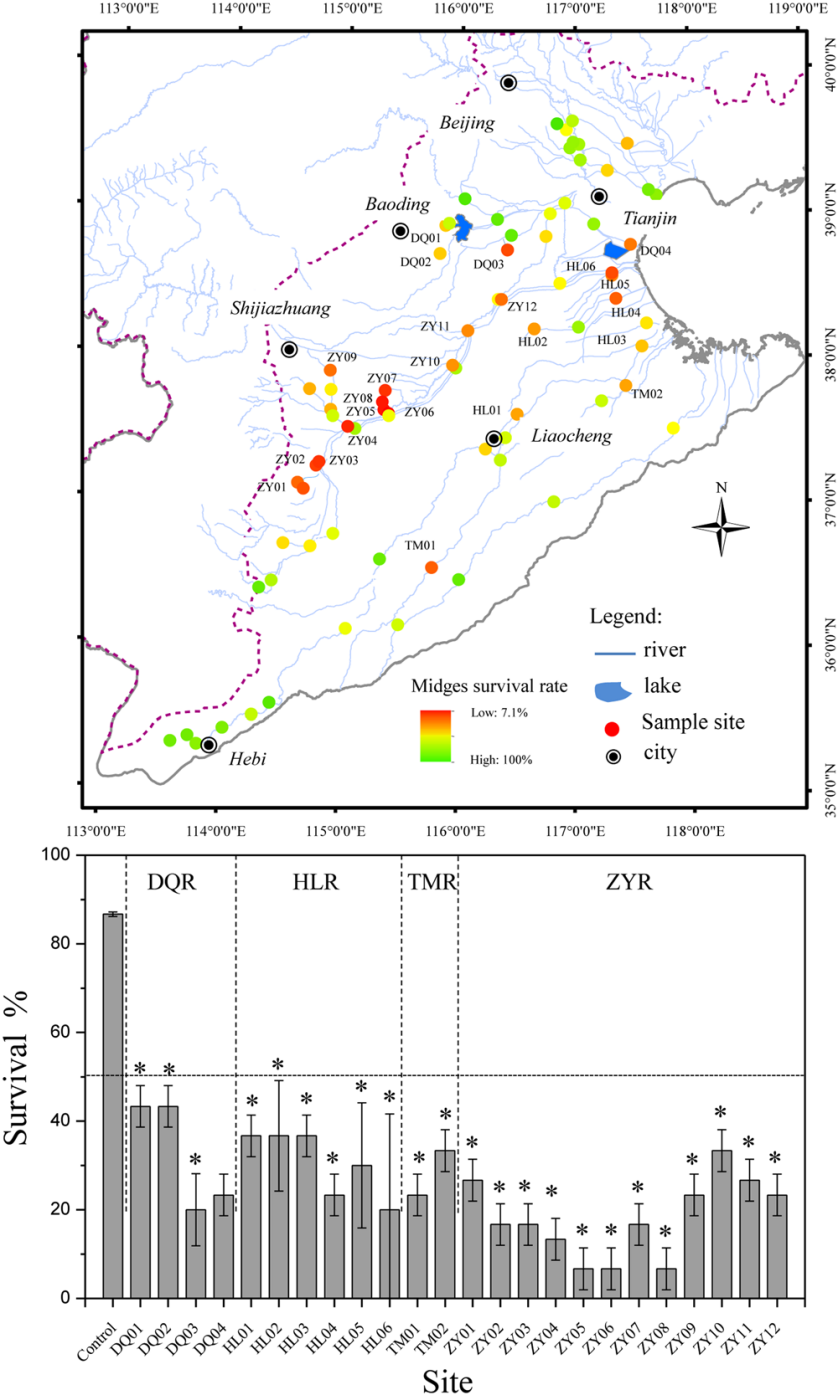
Observed survival in Haihe River sediments in 10-d *Chironomus dilutus* toxicity tests. The mean (±standard deviation) of three replicates was used for each site sediment in the bar graph. Stars indicate significant differences (p < 0.05) between the site sediment and the control. DQR is the Daqing River, HLR is the Heilong River, TMR is the Tuma River, ZYR is the Ziya River. The map was created using ESRI ArcGIS 10.1 (http://www.esri.com/).

The initial toxicity tests of sediment samples showed that most of the samples exhibited high acute toxicity, as showed in Fig. 1. The 10-d survival rates of *Chironomus dilutus* in all samples ranged from 7.1% to 100%, with a mean survival rate of 56%. The survival rates were lower than 50% in samples from more than 24 sites. The most hazardous sites were mainly distributed in the Ziya tributary, in the middle of the Haihe River system, and along the estuary. The mortality of *C. dilutus* was more than 90% at three sites in the Ziya tributary.

The high toxicity of the sediments to benthic organisms in these areas has been noted in previous studies^23, 24^. The biodiversity in the Ziya tributary is low, and previous studies reported only three classes, insecta, gastropoda, and oligochaeta^24^. Yan, *et al*.^23^ reported that there was a significant correlation between contaminants and benthic diatoms in the Haihe Estuary. Studies have reported that these areas were seriously polluted by ammonia nitrogen^25^, heavy metals^26, 27^, and organic compounds^28, 29^. With this background, 24 samples with survival rates lower than 50% were chosen for the subsequent TIE experiments.

### Identification of major toxic classes by TIE in the sediments

**Figure 2:**
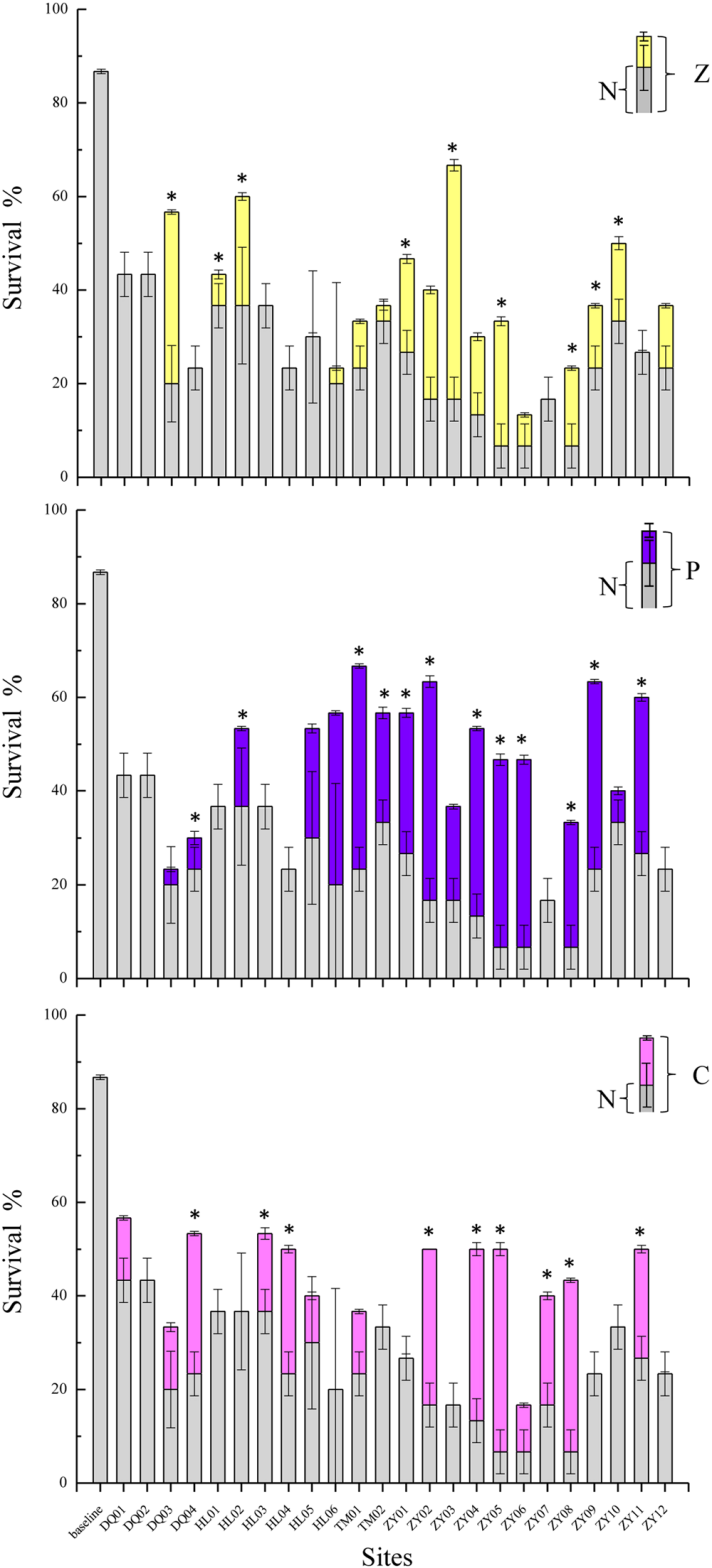
Survival rates of *C. dilutus* in TIEs Phase I. (N = no addition, Z = add zeolite, P = add powdered coconut charcoal, C = add cation-exchange resin. Stars indicate significant differences (p < 0.05) between the site sediment and the control).

The screening toxicity test indicated that heavy metals, ammonia nitrogen, and organic matter were the main toxicants. To determine the cause of the observed mortality to the midges, the sediments were manipulated with three materials, zeolite, cation resin, and coconut charcoal (Fig. 2). In general, additions of zeolite, coconut charcoal, and cation resin increased the survival of *C. dilutus* by 67%, 71%, and 58%, respectively. After additions of zeolite, *C. dilutus* survival increased to more than 50% for samples DQ03, ZY03, and ZY10, located in the middle reaches of DQR and ZYR, which implies that ammonia nitrogen (un-ionized ammonia) played an important role in the mortality caused by these sediments. Additions of coconut charcoal significantly increased the survival rate of the midges in the Ziya and Tuma Rivers and in the estuary, which suggests that organic toxicants contributed to the mortality in these regions. Meanwhile, the toxicity of sediments in the Daqing River (DQ01, DQ04) and in the middle of the Ziya River (ZY07, ZY08) was significantly reduced by adding cation resins. There was more than one toxicant in the sediment samples, which contrasts with the results from previous studies in North America and Europe^20^. For example, the three additives affected midge mortality in samples from sites TM01, ZY02, ZY04, ZY05, and ZY08, which are located in lower reaches of the city. The sediments in the Haihe River were therefore exposed to contamination from multiple sources, and ammonia was the main toxicant, which contrasts with the findings of other studies^13, 20^.

**Figure 3:**
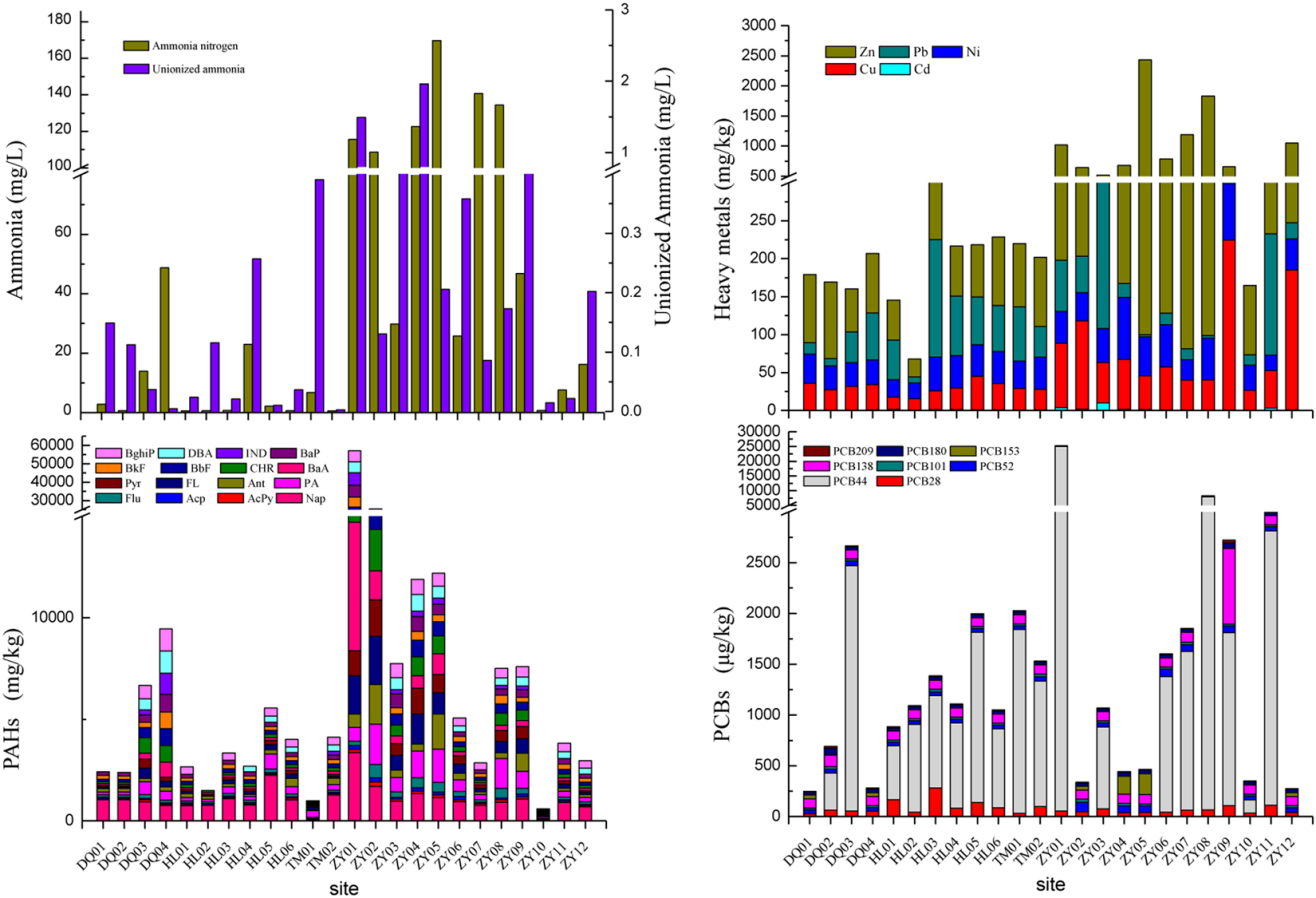
The contents of ammonia, heavy metals, PAHs, and PCBs in sediments.

We used physico-chemical analysis to identify the contaminants of potential concern in the sediment samples. The sediments were mainly comprised of silt, and the total organic carbon (TOC) in Haihe River sediments ranged from 0.25% to 7.42% (Supplementary Table S2). The TOC concentrations were higher at the sampling sites in the Ziya River than in the other tributaries, and were low in the estuary. The concentrations of ammonia nitrogen in interstitial water and in sediment ranged from 0.595 to 170 mg/L, and from 16.5 to 1135 mg/kg dry weight (d.w.), respectively (Fig. 3 and Supplementary Table S2). The highest concentrations were not found at the same site but at adjacent sites; the highest concentration in interstitial water was found at ZY05 while the highest concentration in sediment was found at ZY04.

The concentrations of Cd, Cu, Ni, Pb, and Zn ranged from 0.11 to 9.86 mg/kg, 15.4 to 224 mg/kg, 20.6 to 81.7 mg/kg, 2.79 to 256 mg/kg, and from 23.5 to 2333 mg/kg d.w., respectively (Fig. 3 and Supplementary Table S3). The sites with the highest concentrations were distributed throughout the middle reaches of the Ziya River (ZY03 to ZY09).

Sediments were analyzed for 16 PAHs, and all 16 PAHs were detected in most of the sediments (Fig. 3 and Supplementary Table S4). The concentrations of total PAHs ranged from 590 (ZY10) to 57068 ng/g (ZY01), with a mean value of 7938 ng/g d.w. The mean concentrations of low molecular weight PAHs (2, 3-rings), mid molecular weight PAHs (4-rings), and high molecular weight PAHs (5, 6-rings) were 403, 517, and 575 ng/g, respectively. Several PCB compounds were detected (see Fig. 3 and Supplementary Table S5), and the ∑PCBs ranged from not detected to 25029 ng/kg (ZY01) and had a mean value of 2732 ng/kg d.w. The concentrations of PAHs and PCBs are similar to those reported in the literature^30–32^.

### The contributions of different pollutants calculated by TU in the sediments

The toxicity contributions of ammonia nitrogen, heavy metals, and organic pollutants were evaluated using the toxic unit (TU) approach (Table 1). With the exception of ZY01, ZY03, and ZY04, values of the ammonia-predicted TU by LC_50_ exceeded 1 in sediment samples, which indicates that ammonia nitrogen may have caused the observed toxicity effects. The results of Phase I also indicate that survival of *C. dilutus* increased after zeolite was added to the samples from sites ZY01, ZY03, and ZY04. However, the TU values predicted for several samples were low, and the *C. dilutus* survival also increased when the sediments were treated with zeolite. For example, the survival rate in samples from ZY05 and ZY08 increased from 6.7% to 33.3% and 23.3%, respectively. Ammonia toxicity in aquatic organisms is generally believed to mainly result from non-ionic ammonia. The content of non-ionic ammonia in the interstitial water is related to the solution pH and the total ammonia in the sediment^10^. Therefore, the  criteria maximum concentration (CMC)  was chosen as a benchmark to assess the toxicity of ammonia. The TU values calculated by CMC were greater than 1 in more than 50% samples, which indicated high toxicity in the HRB. The ammonia pollution in ZYR was serious, and the TUs were higher than 1 in nearly all the samples. The evaluation results of ammonia TUs based on CMC were consistent with the change of the C. dilutus survival before and after adding zeolite in sediments. In addition, the toxicity of ammonia may be underestimated because of the loss of ammonia in the overlying water caused by using renewal methods. Accordingly, the toxicity contribution of ammonia in sediment should be paid attention. Furthermore, the toxicity of ammonia was affected by many parameters and the evaluation criteria should be chosen carefully.

**Table 1:** Sum of the Observed Toxic Units (OTU) and the Predicted Toxic Units (PTU) for ammonia, heavy metals (Cd, Cu, Ni, Pb, and Zn), total PAHs, and PCBs.

Sites	OTU	TU-ammonia(LC50)	TU-ammonia(CMC)	TU-Cd	TU-Cu	TU-Ni	TU-Pd	TU-Zn	TU-∑Metals	TU-∑PAHs	TU-∑PCBs
DQ01	**1.13**	0.281	0.636	0.012	0.139	0.072	0.062	0.064	0.349	0.451	—
DQ02	**1.13**	0.212	0.392	0.012	0.106	0.059	0.038	0.072	0.288	0.650	0.001
DQ03	**1.60**	0.070	0.503	0.008	0.124	0.059	0.162	0.041	0.394	**2.997**	0.004
DQ04	**1.53**	0.009	**1.269**	0.037	0.130	0.062	0.249	0.056	0.534	**2.203**	—
HL01	**1.27**	0.046	0.108	0.054	0.063	0.044	0.210	0.038	0.408	0.483	0.001
HL02	**1.27**	0.218	0.404	0.004	0.060	0.040	0.032	0.017	0.153	**1.256**	0.002
HL03	**1.27**	0.040	0.102	0.049	0.097	0.084	0.624	0.057	0.910	0.518	0.002
HL04	**1.53**	0.485	**1.604**	0.050	0.110	0.081	0.316	0.047	0.605	0.145	0.002
HL05	**1.40**	0.020	0.096	0.039	0.173	0.078	0.253	0.049	0.592	**1.344**	0.003
HL06	**1.60**	0.069	0.155	0.038	0.136	0.080	0.244	0.064	0.562	0.432	0.002
TM01	**1.53**	0.737	**1.639**	0.019	0.111	0.068	0.290	0.059	0.547	0.190	0.003
TM02	**1.33**	0.006	0.028	0.030	0.106	0.080	0.164	0.065	0.445	**1.247**	0.002
ZY01	**1.47**	**2.814**	**8.844**	0.146	0.331	0.079	0.273	0.587	**1.416**	0.891	0.036
ZY02	**1.67**	0.247	**3.309**	0.067	0.454	0.070	0.194	0.313	**1.099**	0.481	—
ZY03	**1.67**	**1.046**	**2.935**	0.375	0.209	0.084	**1.031**	0.111	**1.811**	0.507	0.002
ZY04	**1.73**	**3.702**	**10.852**	0.056	0.257	0.155	0.073	0.366	0.907	0.305	0.001
ZY05	**1.87**	0.388	**5.174**	0.046	0.174	0.097	0.011	**1.666**	**1.994**	0.333	0.001
ZY06	**1.87**	0.675	**2.071**	0.020	0.222	0.106	0.061	0.471	0.879	0.148	0.002
ZY07	**1.67**	0.163	**3.954**	0.017	0.154	0.051	0.058	0.792	**1.073**	0.178	0.003
ZY08	**1.87**	0.327	**4.137**	0.016	0.156	0.104	0.014	**1.237**	**1.527**	0.245	0.012
ZY09	**1.53**	0.796	**2.870**	0.018	0.876	0.140	0.111	0.237	**1.382**	0.302	0.004
ZY10	**1.33**	0.028	0.077	0.011	0.103	0.063	0.055	0.065	0.296	0.050	0.001
ZY11	**1.47**	0.041	0.283	0.122	0.193	0.039	0.644	0.141	**1.138**	0.320	0.004
ZY12	**1.53**	0.382	**1.214**	0.030	0.720	0.077	0.086	0.574	**1.487**	0.673	—

The predicted TUs of heavy metals in sediments show that metals made a significant contribution to toxicity at major sampling sites, especially in the Ziya River. Only a few metals showed high toxicity individually. The contents of Zn and Pb at some sites (e.g., ZY03, ZY05, ZY08) in the Ziya River exceeded the LC50 value, which indicates that they were harmful for benthic organisms. The TIE Phase I results indicate that the toxicity of heavy metals decreased after cation resins were added to most samples. However, even though the predicted TU values were high at some sampling sites, the *C. dilutus* survival only increased slightly; for example, the predicted values of the TU of ∑metals were 1.811, 1.382, and 1.487 at sites ZY03, ZY09, and ZY12, respectively. The toxicity of heavy metals in sediments is reflected not only by their total contents; their chemical forms, acid-volatile sulfide sediment concentrations, and interstitial water hardness may also affect their bioavailability.

Among the organics determined, persistent organic pollutants contributed little to the mortality of midges. The predicted TU values for PAHs exceeded 1 at only 5 sampling sites (DQ03, DQ04, HL02, HL05, and TM02). All of the predicted TU values for PCBs were less than 1, which implied that the toxicity from PCBs to the midges was minimal. The TUs of the majority were low, but organics made an obvious contribution to the toxicity of these sediments in the Phase I testing. These conflicting results showed that, apart from PAHs and PCBs, there were other organic toxic pollutants present. For example, previous studies have reported potential ecological risks from organochlorine pesticides (OCPs)^33, 34^, and antibiotics^35, 36^ in the Haihe River basin. Sediments are often contaminated with complex mixtures of toxicants, but unfortunately it is not possible to analyze all the chemical compounds, especially the organic compounds. We plan to focus on the accurate determination of organic toxic substances in a future study. Effect-directed analysis (EDA) is considered a promising tool for the identification of organic toxicants in complex mixtures^37^, and may be used in combination with the TIE approach to improve our toxicity identification capacity^38^.

The results of the TU and TIE toxicity tests show that sediments in the Haihe River have been exposed to multiple contaminants, including ammonia, heavy metals, and organic toxicants. The complex mixture of pollutants is the result of a variety of pollution sources. Increases in the quantity of freshwater consumed and decreases in rainfall have resulted in water shortages in the Haihe River, and sewage and wastewater have become the main sources of water for the Haihe River. Urban sewage, and wastewater from agricultural livestock and poultry have frequently been associated with ammonia; untreated industrial wastewater is the main contributor to heavy metals and organic contaminants. In addition, the Haihe Basin is the most atmospherically polluted area in China, and perhaps in all of Asia^39, 40^, and atmospheric deposition is a major source of contaminants, such as PAHs. Hydrological characteristics also contribute to the heavy pollution of sediments in the Haihe River. There are about 480 dams in the mountainous and plain areas of the Haihe River Basin that alleviate floods, and provide agricultural and landscape irrigation. The numerous dams have restricted hydrological connectivity, decreased the flow velocity, and have ultimately increased the deposition of pollutants. The highly toxic sediment threatens the ecological health of the water, and exacerbates the aquatic ecology degradation. Meanwhile, the complex nature of the pollution in the sediments has increased the challenge faced by the government in managing the water environment.

### The bioavailability of key pollutants (heavy metals) in the sediments

**Figure 4:**
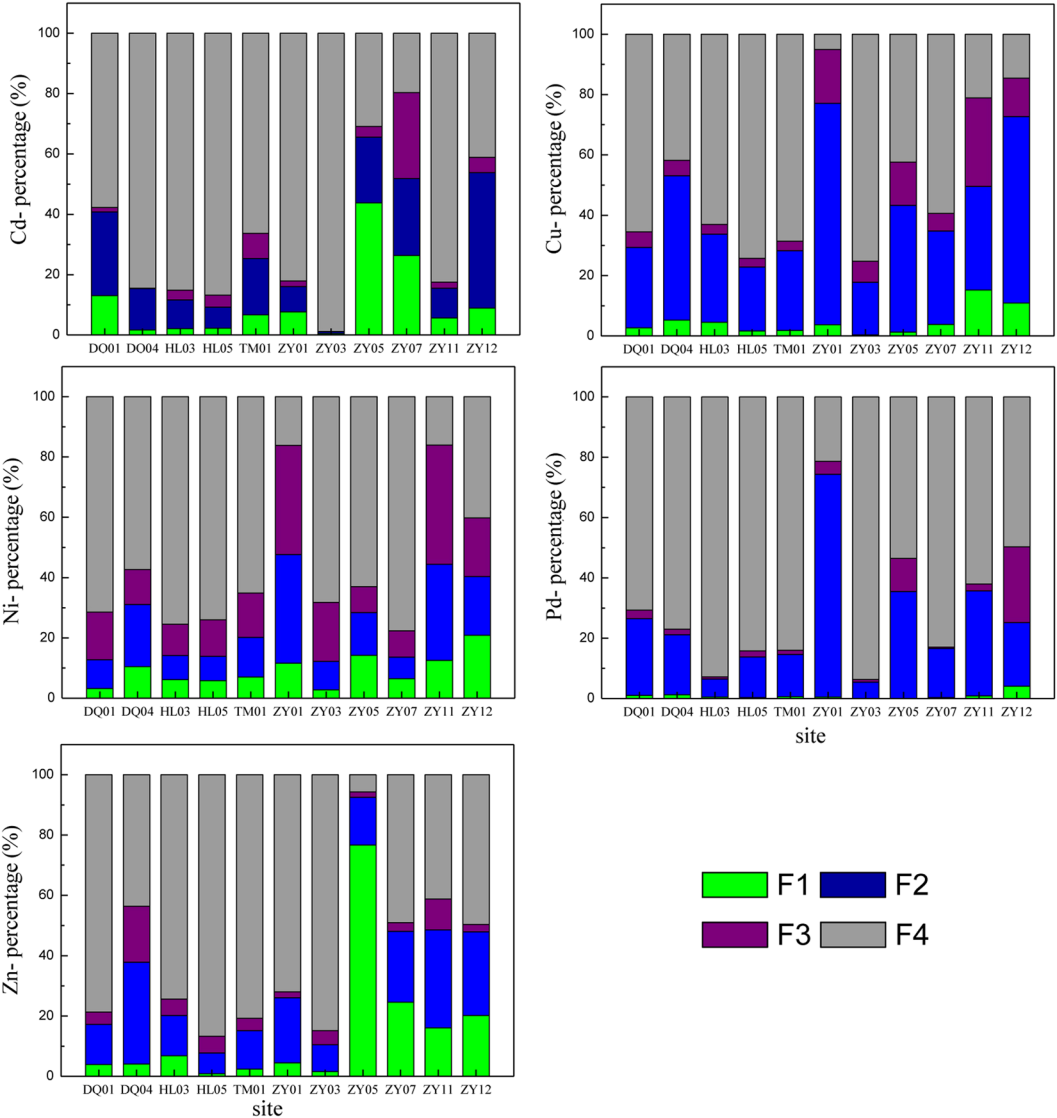
Percentages of the total Cd, Cu, Ni, Pb, and Zn concentrations found in the four chemical fractions F1–F4 in the sediment samples in the Haihe River. (F1 is the water- and acid-soluble fraction, F2 is the reducible fraction, F3 is the oxidizable fraction, and F4 is the residual fraction).

The calculated TUs indicate that metals made a considerable contribution to sediment toxicity at most sites. The total metal content of sediment is a poor indicator of metal toxicity; rather, the toxicity varies according to the chemical form of the metal. In recognition of this, we analyzed samples from 11 representative sites with a modified three-stage BCR sequential extraction procedure that separated the metals into 4 fractions^41^. The concentration of the acid-soluble fraction was regarded as the bioavailable content^42^. The concentrations and percentages of the individual fractions to the total contents are included in Supplementary Table S6 and Fig. 4, respectively.

Overall, the heavy metals in the Haihe River sediments were mainly dominated by the residual fractions. The average concentrations of the residual fractions of Cd, Cu, Ni, Pb, and Zn were 66.8%, 48.3%, 56.8%, 70.2%, and 60.6%, respectively. The characteristics of the acid-soluble fraction varied among elements and tributaries, and, as shown in Fig. 4, the acid-soluble fractions of Cu, Ni, and Pb were below 30% (0.07–20.9%) at all study sites, which shows that the bioavailability of these three metals was limited. The results are consistent with the earlier predicted TU values. At sites ZY05 and ZY12, Cd and Zn were potentially toxic and had relatively high acid-soluble fractions (24.6–76.7%). These results agree with those reported by Tang, *et al*.^27^ for sediments collected from the entire Haihe Basin. Because the farmers along the river practice traditional irrigation with river water, Cd and Zn may accumulate in relatively large amounts in edible portions of crops and may be harmful for human health^43^. Therefore, it is important that heavy metal contaminants, especially Cd and Zn, continue to be monitored in sediments from the Haihe River.

## Materials and Methods

### Study area and sediment sampling

**Figure 5:**
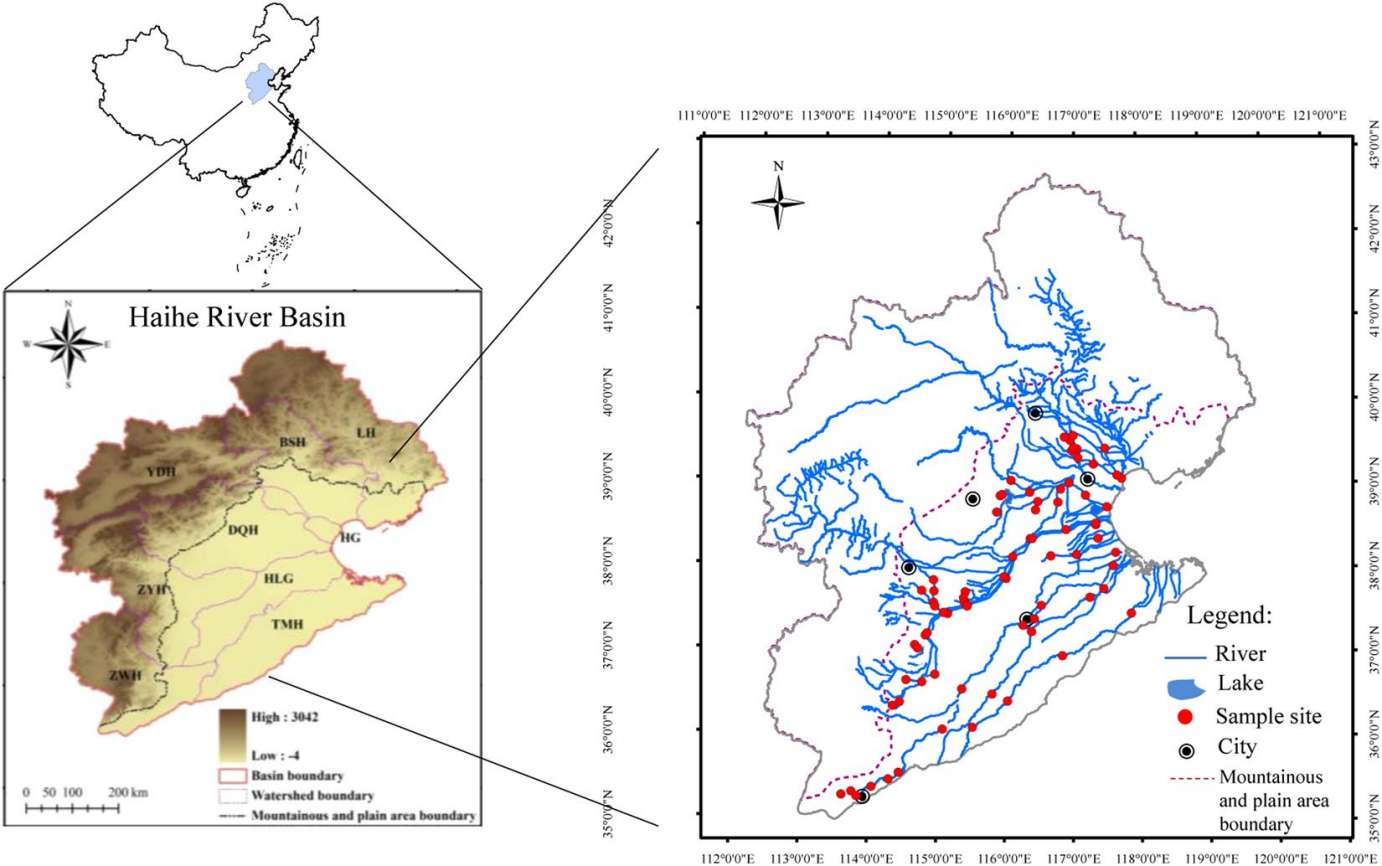
Locations of the sampling sites in the Haihe River Basin, China. The maps were created using ESRI ArcGIS 10.1 (http://www.esri.com/).

The Haihe River Basin in northern China is one of the most important political, economic, and cultural centers in China. Including the highly-developed cities of Beijing and Tianjin, there are more than 20 cities distributed throughout this basin. The watershed covers an area of about 31.8 × 10^4^ km^2^ hills and plateaus occupy almost 60% of the total area, and plain areas account for the remaining 40%^26^. Nine of the main tributaries are distributed into “sectors”, all of which eventually flow into the Bohai Sea (Fig. 5). In the southern part of the Haihe Basin, the main tributaries are the Daqing (DQR), Ziya (ZYR), Heilonggang (HLR), Tuma (TMR), and Zhangwei (ZWR) Rivers. Previous studies have reported that the sediments in the Haihe River are seriously polluted by heavy metals and organic compounds, especially in the plain area of the basin^27, 31, 44, 45^.

We collected a total of 76 sediment samples in the plain area of the Haihe Basin in July 2014 at the sampling sites showed in Fig. 5. At each point, we removed the top 5 cm of sediments with a Van Veen grab sampler. The sediments were then passed through a 2-mm sieve to remove rocks, debris, and benthic organisms^13^. We collected subsamples for grain size, metal content, and toxicity bioassay analysis (including TIE procedures) in polyethylene bottles, and samples for organic matter content and organic compound (PAHs and PCBs) analysis in glass jars^7^. Sediments for toxicity testing and chemical analysis were analysed as soon as possible and stored at 4 and −20 °C, respectively.

### Screening toxicity testing

Based on the recommendations of the US Environmental Protection Agency (US EPA), we chose *C. dilutus*, a common species in the study area, as the target species for the TIE toxicity test^10^.

The procedures followed the U.S.EPA^10^ recommendations. Ten midges were exposed to a 30 mL sediment sample that was covered with 60 mL water in a 120 mL glass beaker. An automatic water exchange system was used to renew the overlying water twice a day. The midges in each beaker were fed daily with 1.5 mL TetraFin® goldfish food (6 mg of dry solids). The tests were performed in triplicate at 23 °C under a 16 h:8 h light:dark photoperiod of ambient laboratory light. Temperature was measured daily; DO, pH, and conductivity were measured three times per week, and ammonia, alkalinity, and hardness were measured twice during the 10-d experiment. At the end of the experiment, the mortality of *C. dilutus* was calculated by counting the organisms sieved from the sediment.

A sample was considered toxic when (a) there was a statistically significant difference between the control and the test samples, and (b) the difference was greater than 20%^2, 7^.

### TIE procedures

#### TIE phase I: Characterization

Cation resin, zeolite, and coconut charcoal were added to the sediments to remove heavy metals, ammonia nitrogen, and organic matter, respectively. After a series of pre-experiments, each sediment sample was mixed with 20% (v/v) of either zeolite or cation resin, and 5% (v/v) of coconut charcoal. We followed the U.S.EPA^10^ procedures for preparing the additives. The zeolite was washed with deionized (DI) water three times before use. The cation exchange resin was rinsed with DI water three times before use, and then was combined with four parts of saline water (sodium chloride 30 g/L), and stored at 4 °C in the dark for 24 hours before use. The coconut charcoal was hydrated with DI water under vacuum for at least 18 hours to remove the air bubbles, and then was centrifuged to remove excess water before use. Meanwhile, the control sediment was manipulated in the same way as the test sediments and was included as treated and untreated controls. The manipulated sediment samples were thoroughly mixed and equilibrated for 24 h before adding the midges^10^. The manipulated sediments were tested in triplicate. The conditions for the 10-d bioassays in the Phase I TIE testing were similar to those in the toxicity screening tests discussed earlier (Screening toxicity testing).

#### TIE Phase II: Identification

Samples were also analyzed for chemicals of concern, including ammonia, metals, and organic pollutants, to identify the contaminants responsible for the observed toxicity (Supplementary Table S2–S5). The analytical methods for ammonia, metals, and organics are presented below.

*Ammonia*. The ammonia concentrations in sediment interstitial water were determined immediately after sediment collection. Wet sediment was thoroughly mixed, and 100 g of sediment was subsampled and centrifuged at 4000 g for 30 min to obtain interstitial water. After filtration, the total ammonia concentration in interstitial water was determined by flow injection analysis (FIA) using conductometric detection^8^. The combined ammonia concentration was calculated from the total ammonia concentration, pH, and water temperature using the compensation formula^46^.

*Metals*. For total trace metal analysis, the samples were microwave-digested with a 5:1 mixture of hydrofluoric and perchloric acids in Teflon vessels (MARS Xpress, CEM). Inductively coupled plasma mass spectrometry (ICP-MS, 7500a; Agilent, USA) was used to measure the cadmium (Cd), copper (Cu), nickel (Ni), lead (Pb), and zinc (Zn) contents. A certified reference material GBW07401, obtained from the Chinese Environmental Monitoring Center, was used as the standard for elemental analysis. More details on sample preparation and metal quantification are presented in the Supplemental Data.

*Organics*. Sediments were analyzed for a suite of organic contaminants comprising 16 PAHs and 28 PCBs that had been previously detected in sediments in the study area. Accelerated solvent extraction (ASE300, Dionex) was used to extract the total contaminant contents of the sediment. Extracts were purified by solid-phase extraction cartridges, and analyzed by gas chromatography-mass spectrometry with internal calibration. More details on sample preparation and chemical analysis are provided in the Supplementary Information (a).

### Toxic Unit (TU) Evaluation

The observed and predicted TU values were used to assess the contribution of each contaminant^21^. Observed TUs and predicted TUs were calculated using Equations (1) and (2):

1 $$\begin{array}{cc}Observed & TU=\frac{OPM}{50}\end{array}\times DF$$

2 $$\begin{array}{cc}Predicted & TU=\frac{Ci}{LC50}\end{array}$$

The observed TUs were derived from the mean mortality of midges exposed to the sediment samples (Equation 1), where OPM was the observed percent mortality, and DF was the dilution factor used for testing all the sediments. In this study, the sediments were not diluted, and so the DF was 1. The predicted TUs represented the sediment toxicity estimated from the chemical contents of the sediments (Equation 2). The individual predicted TU was calculated by dividing the contaminant content (Ci) in sediment by the lethal content 50 (LC50) values acquired from published literature for midges. Not all LC50 values were available, so the sediment quality guidelines (SQGs) were used as the LC50^12, 13, 21^. Ammonia toxicity in aquatic organisms is generally believed to mainly result from non-ionic ammonia. The LC50 value of ammonia was obtained from Besser, *et al*.^47^, the CMC for porewater-ammonia which was suggested by USEPA^48^ was calculated also as the benchmark to evaluate the toxicity of the un-ionized portion of ammonia to midges. The consensus-based probable effect concentrations (PEC) for freshwater sediments were used for total PCBs^49^, and the equilibrium partitioning sediment benchmarks were used for PAHs^50^. The LC50 values for the five metals (Cd, Cu, Ni, Pb, and Zn) were obtained from Shen, *et al*.^51^.

### Statistical analysis

Bioassay results were analyzed with two-way analysis of variance (ANOVA) in SPSS 18.0 software to identify significant differences between control sediments and test samples and for TIE procedures. A significance level of 0.05 was applied.

## Electronic supplementary material

SUPPLEMENTARY INFO
