# Supplementary material for: Using Chironomus dilutus to identify toxicants and evaluate the ecotoxicity of sediments in the Haihe River Basin
Source: Sci Rep. 2017 May 3;7:1438. doi: 10.1038/s41598-017-01631-5 (PMC5431094; doi:10.1038/s41598-017-01631-5)
Supplement: Supplementary file 1 — SUPPLEMENTARY INFO [file 41598_2017_1631_MOESM1_ESM.pdf]

Using *Chironomus dilutus* to identify toxicants and evaluate the ecotoxicity of sediments in the Haihe River

Xiaolei Zhu [1, 2], Baoqing Shan *, [1, 2], Wenzhong Tang *, [1, 2] & Chao Zhang [1, 2]

1. State Key Laboratory of Environmental Aquatic Chemistry, Research Center for Eco-Environmental Sciences, Chinese Academy of Sciences, Beijing 100085, P.R. China

2. University of Chinese Academy of Science, Beijing 100049, P.R. China

* Correspondence and requests for materials should be addressed to B.Q.S. (bqshan@rcees.ac.cn) or W.Z.T. (wztang@rcees.ac.cn).

METHODS

Sediment samples were analyzed for ammonia, metals and organic contaminants, and the respective methods for analyzing the contaminants of concern are discussed below.

Ammonia. Concentrations of ammonia in sediment interstitial water were analyzed immediately on sediment collection. The total ammonia concentration in interstitial water was analyzed by flow injection (FIA) using conductometric detection [1]. The unionized ammonia concentration was calculated from the total ammonia concentration, pH, and water temperature using the compensation formula [2].

Metals. For total trace metal analysis, the samples were microwave-digested with a 5:1 mixture of hydrofluoric and perchloric acids [3] in Teflon vessels (MARS Xpress, CEM). All of the above solutions were stored at 4 ℃ until analysis. Inductively coupled plasma mass spectrometry (ICP-MS, 7500a; Agilent, USA) was used to measure the Cd, Cu, Ni, Pb, and Zn. Certified reference material GBW07401, obtained from the Chinese Environmental Monitoring Center, was used for element analysis. Overall, the metal contents of the standard reference materials were within 96%–110% of the certified values.

After filtration and acidification, interstitial waters were analyzed by means of ICP-MS (7500a; Agilent, USA) to determine the concentrations of dissolved metals (Cd, Cu, Ni, Pb and Zn). For interstitial water element analysis, the analytical quality was assured by analyzing a certified reference material (GSBZ50009-88) provided by the Chinese Environmental Monitoring Center. Overall, trace-metal concentrations were found to be within 83–110% of the certified values.

Non-polar organics. After adding the surrogates, total sediment extractions (5g, dried material) were extracted with a dichloromethane acetone mixture (v/v 1:1) in an accelerated solvent extractor (ASE; 2 cycles at 130°C and 1500 psi). The surrogates of PAHs and PCBs were 1, 4-Dichlorobenzene D4 and Decafluorobiphenyl, separately. Cu-powder (activated with 10% HCl and washed 3 times with methanol) was added for removal of sulphur [4]. After removing the residue water by anhydrous Na_2SO_4, the extract was divided into two equal portions by weight and concentrated to 1 mL for further cleanup[5].

The extract was cleaned using improved Florisil SPE column (1000 mg /6 mL, Agilent, USA). The column was packed with Florisil, 700 mg of activated silica gel and 1 g of anhydrous Na_2SO_4 from the bottom to the top [6]. After conditioning the column with hexane and loading the sample, 17mL of a mixture of hexane and acetone (19:1, v/v) were added to the column as elution solutions in turn. The volume of eluent reduced under a gentle N2 stream to 1 ml.

The PAHs were analyzed on an Agilent 6890-5973 GC-MS in electron impact (EI) mode. The analytes were separated on an Agilent HP-5MS column (30 m × 0.25 mm, film thickness 0.25 μm). Helium was used as carrier gas and pulsed splitless injection was performed with injector temperature at 280 °C. The oven temperature was initiated at 50 °C, held for 3 min, heated to 150 °C at 25 °C/min, held for 3 min, then heated to

240 ^{o}C at 6 ^{o}C/min, then heated to 280 ^{o}C at 16 ^{o}C/min, and held for 10 min. The PCBs were analyzed at 280 ^{o}C for injector temperature. The initial oven temperature for analyzing PCBs was set at 40 ^{o}C, heated to 280 ^{o}C at 20 ^{o}C/min, held for 5 min.

For all of the chemical analyses, quality assurance and quality control were monitored by processing blank and reference standard materials. A calibration standard was analyzed every 10 samples to check the stability of the instruments and ensure the variability of each analyte was within 20%. Surrogate standards were added to each sample before extraction to check the whole procedure of sample preparation. The recoveries of metals in spiked samples were 71–109%. The d10-acenaphthene, d12-chrysene, d12-perylene, d10-phenanthrene and 2,4,5,6-Tetrachloro-m-xylene, added before the extractions of sediments, were applied as internal standards. Further quality control measures included the analysis and monitoring of procedural blanks, recovery of spiked compounds and other performance characteristics (e.g. precision, LODs and LOQs). The LOD for PAHs and PCBs in biota was 1-5 ng/g, dw.

Table S1 Modified BCR sequential extraction scheme [7]

step	Target phase	Extraction agent and procedures
**1**	Acid soluble fraction	Duplicate samples, 0.1g+20ml of 0.11Mm CH_3COOH, Shaking time 10h and centrifuge
**2**	Reducible fraction	Step 1 residue sample, 20 ml NH_2OH-HCl 0.5Mm (pH=1.5), Shaking time 10h and centrifuge
**3**	Oxidizable fraction	Step 2 residue sample, 5ml H_2O_2 (8.8Mm) heat to 90°C for1h (Repeated once) and then add 20ml CH_3COONH_4 1Mm (pH=2), Shaking time 10h and centrifuge
**4**	Residual fraction	Step 3 residue sample, HNO_3, HCl (1ml:3ml) ,digested 30 min

Table S2 Physicochemical properties of sediments and interstitial water

sites	grain size			TOC（%）	interstitial water			
	Clay (%)	Silt (%)	Sand (%)		pH	Hardness (mg/L)	Ammonia nitrogen (mg/L)	Unionized ammonia (mg/L)
DQ01	33.5	60.1	6.4	1.03	8.12	252	2.84	0.149
DQ02	29.0	55.0	15.9	0.71	8.65	310	0.635	0.112
DQ03	26.0	55.1	19.0	0.32	6.83	2027	13.9	0.037
DQ04	34.3	63.3	2.4	0.53	5.39	1586	48.7	0.005
HL01	16.2	64.0	19.8	0.92	8.01	516	0.595	0.024
HL02	11.1	57.9	30.9	0.25	8.65	519	0.655	0.116
HL03	22.6	64.0	13.4	1.13	7.84	648	0.770	0.021
HL04	23.9	70.3	5.9	3.13	7.45	642	23.0	0.257
HL05	29.7	66.4	3.8	0.77	7.09	677	2.14	0.010
HL06	32.5	62.8	4.7	1.54	8.15	1004	0.655	0.037
TM01	29.3	67.2	3.5	0.80	8.16	512	6.72	0.391
TM02	22.5	66.8	10.8	0.55	7.11	820	0.610	0.003
ZY01	14.5	66.5	18.9	7.42	7.51	439	116	1.491
ZY02	21.2	75.2	3.5	7.03	6.48	713	109	0.131
ZY03	13.3	65.3	21.5	2.19	7.67	620	29.8	0.554
ZY04	17.4	76.4	6.2	5.66	7.60	634	123	1.962
ZY05	12.6	49.0	38.4	5.44	6.48	838	170	0.205
ZY06	6.0	43.3	50.7	5.49	7.54	3032	25.8	0.358
ZY07	12.4	59.7	27.9	2.69	6.19	780	141	0.086
ZY08	14.7	72.1	13.1	4.64	6.51	599	134	0.173
ZY09	15.0	74.1	10.9	3.86	7.35	670	46.8	0.422
ZY10	21.8	74.5	3.8	1.68	7.70	853	0.745	0.015
ZY11	16.4	69.3	14.3	1.87	6.86	779	7.64	0.022
ZY12	13.4	55.9	30.8	0.69	7.49	676	16.3	0.202

Table S3 Concentrations of heavy metals in sediments (mg/kg) and interstitial water (μg/L)

sites	Cd		Cu		Ni		Pb		Zn	
	interstitial water	Sediment	interstitial water	Sediment	interstitial water	Sediment	interstitial water	Sediment	interstitial water	Sediment
DQ01	0.856	0.317	7.06	35.7	3.39	38.1	6.26	15.3	90.3	89.9
DQ02	0.842	0.323	8.59	27.1	3.14	31.5	7.41	9.5	126	101
DQ03	0.886	0.212	21.2	31.8	5.36	31.2	9.88	40.2	91.6	56.9
DQ04	0.887	0.976	20.8	33.2	9.26	32.6	6.46	61.7	24.7	78.5
HL01	0.903	1.41	33.2	16.2	10.5	23.2	6.00	52.0	47.4	52.7
HL02	0.927	0.114	12.3	15.4	8.60	21.0	7.08	7.9	85.3	23.5
HL03	0.867	1.28	21.9	24.9	9.67	44.3	6.20	155	86.4	79.6
HL04	0.833	1.32	8.52	28.2	4.63	42.9	6.21	78.4	77.8	65.9
HL05	0.872	1.02	9.48	44.2	8.19	41.5	6.91	62.7	87.1	69.1
HL06	0.841	1.01	6.45	34.7	24.74	42.2	6.41	60.5	100	90.2
TM01	0.943	0.491	9.03	28.5	14.7	35.8	7.58	71.8	70.3	83.2
TM02	0.874	0.794	14.7	27.1	4.70	42.3	6.44	40.8	70.4	91.0
ZY01	0.867	3.83	7.75	84.6	7.22	41.8	8.36	67.8	47.8	822
ZY02	0.554	1.76	20.3	116	66.5	37.3	7.87	48.2	59.0	438
ZY03	0.882	9.86	9.97	53.5	20.3	44.5	6.62	256	122	156
ZY04	0.881	1.47	11.4	65.8	14.9	81.7	7.60	18.1	65.1	512
ZY05	0.856	1.20	10.9	44.4	6.36	51.5	6.96	2.8	138	2333
ZY06	0.860	0.521	9.56	56.8	11.1	55.9	8.48	15.1	115	659
ZY07	0.937	0.455	78.2	39.5	75.3	27.1	17.1	14.3	143	1109
ZY08	0.890	0.430	12.5	39.9	18.0	55.0	7.74	3.5	121	1731
ZY09	1.064	0.468	9.55	224	10.6	74.0	6.86	27.5	53.9	332
ZY10	0.848	0.280	8.79	26.3	6.99	33.3	6.14	13.7	120	91.1
ZY11	0.851	3.20	7.52	49.3	4.86	20.6	6.36	160	70.9	197
ZY12	0.850	0.777	12.0	184	5.32	40.8	6.34	21.4	116	804
LC50		26.3		256		529		248		1400

LC50 were from reference [8,9]

Table S4 Concentrations of 16 PAHs in sediments (μg/kg)

sites	Nap	AcPy	Acp	Flu	PA	Ant	FL	Pyr	BaA	CHR	BbF	BkF	BaP	IND	DBA	BghiP	∑PAHs
DQ01	1034	60	75	112	134	168	83	76	89	90	139	173	180	ND[a]	ND	ND	2413
DQ02	1041	64	63	105	144	178	67	58	85	99	136	170	178	ND	ND	ND	2388
DQ03	939	95	77	190	620	164	511	441	294	774	484	257	368	250	544	662	6670
DQ04	759	63	65	127	451	212	267	203	750	813	826	825	881	1036	1083	1097	9458
HL01	754	63	62	108	164	200	102	87	97	152	156	166	181	ND	ND	368	2660
HL02	776	ND	72	97	118	151	48	44	94	93	ND	ND	ND	ND	ND	BRL	1493
HL03	1092	64	65	137	318	162	173	133	118	124	185	213	195	ND	ND	366	3345
HL04	780	61	63	114	180	107	142	119	117	175	181	174	202	ND	280	ND	2695
HL05	2230	77	74	183	724	212	257	206	134	178	190	180	211	ND	308	394	5558
HL06	1033	83	69	146	360	403	209	181	123	168	182	210	193	ND	275	379	4014
TM01	70	19	18	65	329	39	117	59	23	34	36	24	36	43	44	37	992.9
TM02	1273	66	64	114	270	309	100	90	157	168	194	209	224	197	311	382	4128
ZY01	3362	149	203	207	685	659	1879	1231	6335	6257	5508	5449	6225	6915	5973	6031	57068
ZY02	1696	211	214	656	1988	1955	2365	1801	1435	2036	2395	931	2014	686	2624	2368	25375
ZY03	978	91	88	278	696	368	734	563	388	532	552	294	688	207	591	697	7745
ZY04	1349	117	165	495	1312	346	1475	1280	606	931	826	429	725	274	809	755	11894
ZY05	1147	127	147	464	1643	1741	1040	901	1015	882	698	349	519	292	594	631	12190
ZY06	947	77	170	260	566	332	443	413	200	246	239	260	231	ND	289	393	5066
ZY07	769	81	67	125	255	132	154	186	109	124	149	182	179	ND	ND	353	2865
ZY08	913	126	86	472	1476	280	555	539	263	597	445	439	299	188	360	469	7507
ZY09	1071	117	144	267	836	900	712	604	292	511	389	237	377	187	437	525	7606
ZY10	60	17	17	31	67	23	47	37	24	24	39	37	32	46	48	42	589.95
ZY11	905	64	68	137	286	154	202	155	140	200	230	252	281	ND	340	407	3821
ZY12	687	60	65	125	228	133	103	84	119	135	164	195	199	ND	301	363	2961
ESB	385	491	452	538	596	594	707	697	841	844	979	981	965	1131	1115	1095	

ND[a]=Not detected

ESBs were from reference [10]

Table S5 Concentrations of PCBs in the sediments (ng /kg)

sites	PCB28	PCB44	PCB52	PCB138	PCB153	∑PCBs
DQ01	ND[a]	ND	ND	ND	ND	ND
DQ02	ND	366	ND	113	ND	479
DQ03	ND	2415	ND	ND	ND	2415
DQ04	ND	ND	ND	ND	ND	ND
HL01	165	531	ND	ND	ND	697
HL02	ND	865	ND	ND	ND	865
HL03	281	910	ND	ND	ND	1191
HL04	ND	841	ND	ND	ND	841
HL05	139	1676	ND	ND	ND	1815
HL06	ND	776	ND	ND	ND	776
TM01	ND	1811	ND	ND	ND	1811
TM02	ND	1235	ND	ND	ND	1235
ZY01	ND	24923	106	ND	ND	25029
ZY02	ND	ND	ND	ND	ND	ND
ZY03	ND	808	ND	ND	ND	808
ZY04	ND	ND	ND	ND	177	177
ZY05	ND	ND	ND	ND	208	208
ZY06	ND	1337	ND	ND	ND	1337
ZY07	ND	1562	ND	ND	ND	1562
ZY08	ND	7893	ND	ND	ND	7893
ZY09	108	1702	ND	744	ND	2554
ZY10	ND	129	ND	ND	ND	129
ZY11	111	2700	ND	ND	ND	2812
ZY12	ND	ND	ND	ND	ND	ND

ND[a]=Not detected

PEC was from reference [9]

Table S6 Concentrations in the different chemical fractions F1–F4 for the sediment samples in the Haihe River. (F1 is the water- and acid-soluble fraction, F2 is the reducible fraction, F3 is the oxidizable fraction, and F4 is the residual fraction)

sites	Cd[a]				Cu				Ni				Pd				Zn			
	F1	F2	F3	F4	F1	F2	F3	F4	F1	F2	F3	F4	F1	F2	F3	F4	F1	F2	F3	F4
DQ01	0.041	0.088	0.005	0.183	0.948	9.53	1.86	23.4	1.22	3.63	6.04	27.2	0.525	13.1	1.46	36.2	3.96	13.6	4.22	80.4
DQ04	0.016	0.134	0.001	0.825	1.74	15.9	1.72	13.9	3.41	6.72	3.80	18.7	0.760	12.3	1.14	47.5	3.21	26.5	14.5	34.2
HL03	0.027	0.122	0.042	1.09	1.14	7.27	0.78	15.7	2.72	3.54	4.60	33.4	0.745	9.26	1.11	144	5.42	10.6	4.32	59.2
HL05	0.023	0.069	0.041	0.873	0.553	7.38	0.99	25.8	2.48	3.39	5.12	31.2	0.196	8.13	1.21	51.0	0.752	6.18	5.06	78.2
TM01	0.033	0.091	0.041	0.325	0.522	7.52	0.91	19.5	2.50	4.72	5.28	23.3	0.440	10.0	1.05	60.3	2.02	10.6	3.39	67.2
ZY01	0.294	0.319	0.074	3.14	3.15	62.1	15.1	4.27	4.87	15.1	15.1	6.76	0.323	50.1	2.89	14.5	36.6	178	16.5	592
ZY03	0.047	0.059	0.010	9.75	0.161	9.35	3.70	40.3	1.24	4.18	8.72	30.4	0.187	13.8	2.11	240	2.43	13.9	7.26	132
ZY05	0.003	0.005	0.043	0.372	0.543	18.7	6.38	18.8	7.30	7.31	4.43	32.4	0.002	0.987	0.306	1.49	1790	368	41.3	134
ZY07	0.001	0.001	0.129	0.090	1.52	12.2	2.31	23.5	1.76	1.93	2.34	21.0	0.048	2.33	0.070	11.9	273	261	31.7	544
ZY11	0.180	0.316	0.065	2.64	7.49	16.9	14.5	10.4	2.57	6.57	8.13	3.30	1.34	55.6	3.68	99.0	31.8	64.0	20.2	81.3
ZY12	0.069	0.349	0.039	0.319	20.1	114	23.6	26.7	8.53	7.95	7.95	16.4	0.871	4.51	5.37	10.6	162	222	20.2	399

[a] heavy metal concentrations (mg kg^{-1})

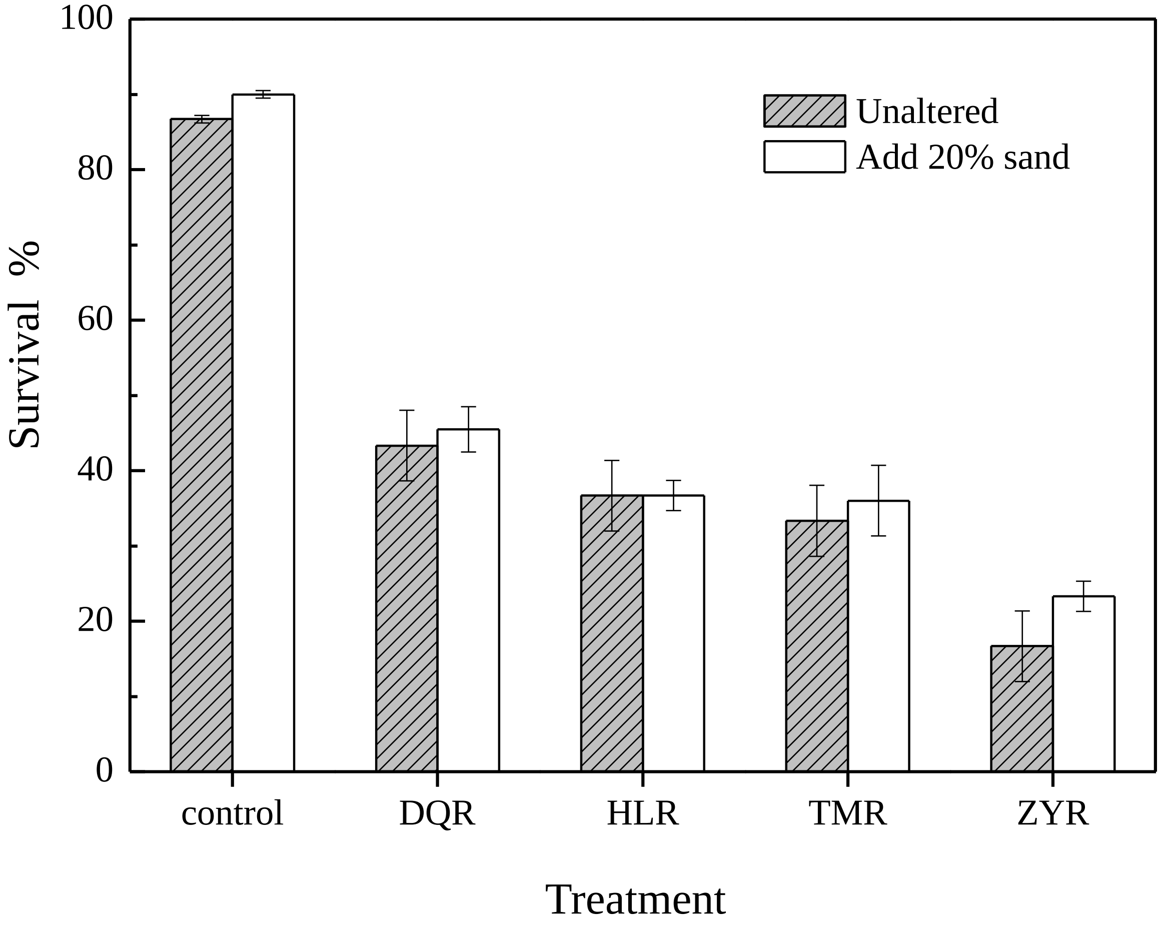

Figure S1 Results of sand Dilution Black Test

Reference

1 Matos, M. D. F., Botta, C. M. R. & Fonseca, A. L. Toxicity Identification Evaluation (Phase I) of water and sediment samples from a tropical reservoir contaminated with industrial and domestic effluents. *Environmental Monitoring & Assessment* **186**, 7999-8006 (2014).

2 Emerson, K., Russo, R. C., Lund, R. E. & Thurston, R. V. Aqueous Ammonia Equilibrium Calculations: Effect of pH and Temperature. *Journal of the Fisheries Research Board of Canada* **32**, 2379-2383 (2011).

3 Tessier, A., Campbell, P. G. & Bisson, M. Sequential extraction procedure for the speciation of particulate trace metals. *Analytical chemistry* **51**, 844-851 (1979).

4 Tuikka, A. I. *et al.* Predicting the bioaccumulation of polyaromatic hydrocarbons and polychlorinated biphenyls in benthic animals in sediments. *Science of the Total Environment* **563**, 396-404 (2016).

5 Yi, X., Li, H., Ma, P. & You, J. Identifying the causes of sediment-associated toxicity in urban waterways in South China: Incorporating bioavailabillity-based measurements into whole-sediment toxicity identification evaluation. *Environmental Toxicology & Chemistry* **34**, 1744–1750 (2015).

6 Wang, D. W. *et al.* Simultaneous Determination of 28 Polychlorinated Biphenyls and 16 Polycyclic Aromatic Hydrocarbons in Sediments Using ASE-SPE-GC-QqQ-MS/MS. *Chinese Journal of Analytical Chemistry* **41**, 861-868 (2013).

7 Arain, M. B. *et al.* Time saving modified BCR sequential extraction procedure for the fraction of Cd, Cr, Cu, Ni, Pb and Zn in sediment samples of polluted lake. *Journal of Hazardous Materials* **160**, 235-239 (2008).

8 Shen, H. *et al.* Bio-Toxicity and bioavailability of metal-spiked freshwater sediments to benthic invertebrates. *Huanjing Kexue Xuebao* **34**, 272-280 (2014).

9 Macdonald, D. D., Ingersoll, C. G. & Berger, T. A. Development and Evaluation of Consensus-Based Sediment Quality Guidelines for Freshwater Ecosystems. *Archives of Environmental Contamination & Toxicology* **39**, 20-31 (2000).

10 Burgess, R. M., Berry, W. J., Mount, D. R. & Toro, D. M. D. Mechanistic sediment quality guidelines based on contaminant bioavailability: equilibrium partitioning sediment benchmarks (ESBs). *Environmental Toxicology & Chemistry* **32**, 102-114 (2013).
